# Takotsubo Syndrome and Oxidative Stress: Physiopathological Linkage and Future Perspectives

**DOI:** 10.3390/antiox14050522

**Published:** 2025-04-27

**Authors:** Alfredo Mauriello, Carmen Del Giudice, Gerardo Elia Del Vecchio, Adriana Correra, Anna Chiara Maratea, Martina Grieco, Arianna Amata, Vincenzo Quagliariello, Nicola Maurea, Riccardo Proietti, Antonio Giordano, Antonello D’Andrea, Vincenzo Russo

**Affiliations:** 1 S.C. Cardiologia, Istituto Nazionale Tumori, IRCCS, Fondazione “G. Pascale”, 80131 Naples, Italy; alfredo.mauriello93@libero.it (A.M.); annachiara.maratea@gmail.com (A.C.M.); v.quagliariello@istitutotumori.na.it (V.Q.); n.maurea@istitutotumori.na.it (N.M.); 2 Cardiology Unit, Boscotrecase Hospital, ASL NA3Sud, 81042 Boscotrecase, Italy; carmen.delgiu93@gmail.com; 3 Cardiology Unit, “A. Guerriero” Hospital, ASL CE, 81025 Marcianise, Italy; delvecchiogerardo@gmail.com; 4 Intensive Cardiac Care Unit, “San Giuseppe Moscati” Hospital, ASL CE, 81031 Aversa, Italy; adrianacorrera@gmail.com; 5 Cardiology Unit, S. Giovanni Bosco Hospital, ASL NA1, 80100 Naples, Italy; m.grieco93@gmail.com; 6 Department of Cardiovascular Medicine, Fondazione Policlinico Universitario A. Gemelli IRCCS, 00168 Rome, Italy; arianna.amata2@gmail.com; 7 Liverpool Centre for Cardiovascular Science at University of Liverpool, Liverpool John Moores University and Liverpool Heart & Chest Hospital, Liverpool L8 7TX, UK; riccardo.proietti@liverpool.ac.uk; 8 Sbarro Institute for Cancer Research and Molecular Medicine and Center of Biotechnology, College of Science and Technology, Temple University, BioLife Science Bldg, Suite 431-1900 N 12th Street, Philadelphia, PA 19122, USA; president@shro.com; 9 Cardiology and Intensive Care Unit, Department of Cardiology, “Umberto I” Hospital, 84014 Nocera Inferiore, Italy; antonellodandrea@libero.it; 10 Cardiology Unit, Department of Medical and Translational Sciences, University of Campania “Luigi Vanvitelli”, Monaldi Hospital, 80131 Naples, Italy

**Keywords:** Takotsubo syndrome, oxidative stress, acute coronary syndrome, heart failure, cancer

## Abstract

Takotsubo syndrome (TTS) is an acute coronary syndrome of unknown prevalence with a physiopathological mechanism that is not yet fully understood. The course is generally benign. Current therapeutic management is based on limited evidence. Oxidative stress seems to play a role in the pathogenesis of cardiovascular diseases, especially regarding the endothelial dysfunction underlying TTS. The present review aims to describe the pathophysiological mechanisms linking oxidative stress and TTS, explore the impact of oxidative stress on TTS, and evaluate the efficacy of anti-oxidative stress therapies on TTS.

## 1. Introduction

Takotsubo syndrome (TTS) is an acute and reversible cardiomyopathy characterized by a clinical presentation similar to acute coronary syndrome (ACS) [1] in the absence of non-obstructive coronary atherosclerotic disease. TTS prevalence accounts for 2–3% of patients presenting with ACS [2] and increases to 5–6% in female patients [3]. A multimodality approach is often needed for the differential diagnosis with other acute cardiac presentations [4]. There are several pathophysiological hypotheses on the underlying mechanisms of TTS; however, the activation of the sympathetic nervous system, triggered by emotional or physical stress, with massive release of catecholamines seems to be the most plausible [5,6]. Oxidative stress is a pathological condition caused by an imbalance between the production and accumulation of oxygen-reactive species (ROS) in cells and tissues [7]. Several pre-clinical data link oxidative stress and cardiovascular diseases [8], while the human data between oxidative stress and cardiovascular disease are poorer. The aim of the present comprehensive review is to describe the pathophysiological link between oxidative stress and TTS and to evaluate the effectiveness of anti-oxidative stress therapies on TTS.

## 2. Diagnosis of Takotsubo Syndrome

A multimodality approach is often needed to diagnose TTS [9]. A stressful trigger, emotional or physical, is typically present in 90% of cases [10]. The 12 lead electrocardiogram (ECG) commonly shows acute dynamic changes at presentation, resembling those of an acute coronary syndrome [11]. The most common abnormalities are ST elevation, T-wave inversion, left bundle-branch block (LBBB), and prolonged QTc interval. ST elevation and T-wave inversion are widespread and not localized to a vascular territory [12]. T-wave inversion and QTc prolongation usually start to resolve after 2–6 days over subsequent weeks or months [13].

Cardiac troponin serum levels are typically raised, although peak values are lower than ST-elevation myocardial infarction (MI) and are more comparable to those of non-ST-elevation MI [14]. The diagnosis of TTS is confirmed by negative or non-obstructive coronary artery disease in an invasive coronary angiogram [15]. Left ventriculography confirms the diagnosis when characteristic ballooning of the left ventricle is detected.

In 50–80% of cases, there is an apical and midventricular dyskinesis, akinesis, or hypokinesis with basal sparing [10]. The pathognomonic pattern of TTS is characterized by circumferential midventricular wall motion abnormality with basal and apical hyperkinesis [16]. Other rarer variants involve only the right ventricle or isolated segments of the left ventricle [17].

Echocardiography is useful to describe the severity and location of wall motion abnormalities and to identify potential complications of TTS (obstruction of left ventricular outflow in about 20% of cases or left ventricular thrombus formation) [18,19].

Finally, cardiac magnetic resonance (CMR) with gadolinium contrast administration is useful in the differential diagnosis of acute myocardial infarction and myocarditis [20]. Differently from these latter conditions, fibrosis by late gadolinium enhancement is not usually a feature of TTS, and myocardial edema is limited to regions with abnormal contractility. It resolves gradually over weeks or months after the index event, typically taking much longer to recover than myocardial contractility [21].

## 3. Pathogenesis of Takotsubo Syndrome

The TTS pathophysiology is not completely understood, and a wide range of pathogenetic hypotheses have been proposed. Most patients develop TTS after intense emotional or physical stressors [22]; therefore, catecholamine seems to play a crucial role in TTS [10,23].

Stressful events lead to the activation of central nervous system (CNS) cognitive centers with stimulation of the sympathetic nervous system (SNS) and an acute release of catecholamines (epinephrine and norepinephrine) by the adrenal medulla [24,25]. The association of TTS with pheochromocytoma and the presence of autonomic nervous system dysfunction among TTS patients strongly support this hypothesis. Moreover, the intravenous administration of catecholamines or beta-agonists reproduces the clinical characteristics of TTS [11,22,25,26]. A significant stressful event can be the diagnosis and experience of cancer. Cancer can lead to an increase in catecholamines and oxidative stress, both directly and indirectly, through pharmacological and surgical treatments, which can contribute to the development of TTS [27,28,29].

**Figure 1:**
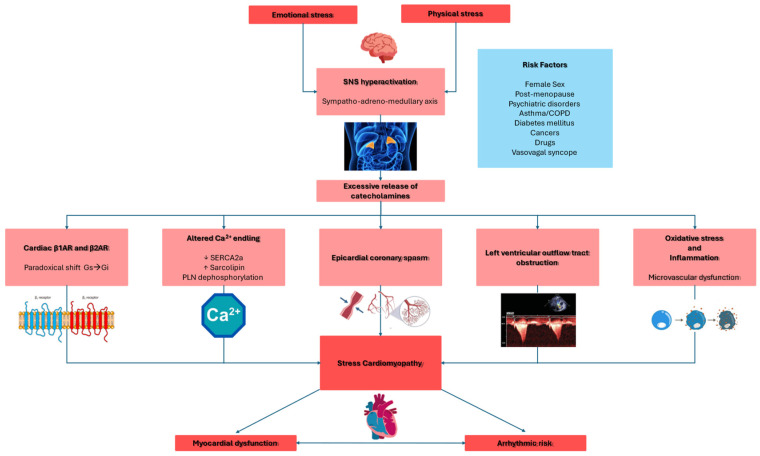
This schematic illustration delineates the pivotal pathogenetic mechanisms implicated in the development of Takotsubo syndrome. β1AR: beta-1 adrenergic receptor; β2AR: beta-2 adrenergic receptor; COPD: chronic obstructive pulmonary disease; Gi: inhibitory guanine nucleotide-binding proteins; Gs: stimulatory guanine nucleotide-binding proteins; LVOTO: left ventricle outflow tract obstruction; PNL: phopsholamin; SERCA2a: sarco-endoplasmic reticulum calcium ATPase 2a; SNS: sympathetic nervous system. Red: principal triggers and targets. Pink: physiopathological and molecular mechanisms. Blue: risk factors.

Catecholamines lead to myocardial dysfunction through several mechanisms (Figure 1), including direct myocardial toxicity [30,31,32], myocardial ischemia caused by epicardial or microvascular dysfunction, as well as “supply/demand mismatch” due to increased myocardial workload [33,34], and left ventricular outflow tract obstruction [35].

Catecholamine-induced myocardial toxicity occurs through β-adrenergic receptor (βAR) activation (β1AR and β2AR) through several mechanisms [32]. Very high levels of epinephrine paradoxically reduce cardiac inotropic by shifting β2AR signaling from the stimulatory protein Gs to the inhibitory protein Gi pathway [32,36,37,38]. In the LV, β1AR and β2AR densities are highest at the apex, making it particularly sensitive to catecholamines, especially epinephrine [10]. Moreover, structural differences, such as reduced caveolae density at the apex, further contribute to heightened catecholamine responsiveness [39]. For these reasons, most TTS occurs with systolic dysfunction at the apical segments.

Finally, β2AR activation leads to peroxynitrite generation—a toxic free radical implicated in myocardial injury [40,41]. Translational research in isoprenaline-treated rats replicating TTS demonstrated a significant increase in 3-nitrotyrosine and poly (ADP-ribose) content, indicating nitro-oxidative and nitrosative stress, as well as activation of poly (ADP-ribose) polymerase-1 (PARP-1). Notably, pretreatment with a PARP-1 inhibitor mitigated negative inotropic effects and reduced nitro-oxidative/nitrosative stress, suggesting that the peroxynitrite/PARP-1 cascade may contribute to negative inotropy in this TTS model [40,42]. The same elevated nitrosative stress markers have been found in myocardial samples from TTS patients [40]. Moreover, the activation of PARP-1 potentially disrupts the synthesis, transport, and utilization of high-energy phosphometabolites such as phosphocreatine (PCr) and adenosine triphosphate (ATP). Indeed, a significant reduction in the PCr/ATP ratio has been observed in patients with TTS using cardiac magnetic resonance (CMR), including cardiac 31P-spectroscopy, suggesting impaired myocardial energy metabolism [43,44,45,46,47].

Several animal models demonstrated that βAR activation induces apical fibrosis, contractile dysfunction, and metabolic alterations, mirroring human TTS [48,49,50,51,52,53]. In patients with TTS, endomyocardial biopsies reveal contraction band necrosis, which is typically observed in catecholamine-excess conditions like pheochromocytoma [54].

Among TTS patients, the increased catecholamine levels cause disruption of calcium homeostasis. In particular, the downregulation of the sarcoplasmic reticulum (SR) calcium pump (SERCA2a) and the upregulation of sarcolipin reduce the calcium reuptake [55]; moreover, the phospholamban dephosphorylation decreases the calcium affinity. Both these mechanisms lead to systolic and diastolic dysfunction [53].

However, not all TTS patients exhibit elevated catecholamine levels. Therefore, SNS hyperactivation and catecholamines surge may trigger TTS, but other mechanisms are required to explain the complexity of the disease.

Clinical analogies between TTS and acute coronary syndrome (ACS) suggest that myocardial ischemia might play a role in the development of myocardial dysfunction [10,23,24,33,56,57].

High levels of catecholamines led to increased chronotropism and blood pressure, resulting in increased afterload and, finally, myocardial oxygen demand. Moreover, catecholamines cause epicardial vasospasm and microvascular dysfunction. The simultaneous multivessel coronary vasospasm, leading to myocardial stunning, is the first proposed mechanism for TTS [33,34]; however, most TTS cases do not show epicardial coronary vasospasm since coronary angiography has shown spontaneous coronary vasospasm only in 5–10% of TTS patients, and induced vasospasm through provocative testing was found in about 28% of cases [58]. Additionally, the presence of LVWMAs, particularly the apical-sparing pattern, does not support coronary spasm as the primary cause of TTS [58].

The hypothesis of microvascular dysfunction as a mechanism underlying TTS [59] is supported by the high percentage of TTS patients with normal epicardial coronary arteries, highlighting the need for alternative methods to assess coronary microcirculation [3]. Several studies based on thrombolysis in myocardial infarction (TIMI) frame count, coronary flow reserve (CFR), and myocardial contrast echocardiography (MCE) showed impaired coronary flow in specific left ventricular segments, which contributes to myocardial dysfunction in TTS patients [56,60,61]. Intravenous adenosine administration transiently improves myocardial perfusion, wall motion, and LVEF, supporting the microvascular hypothesis [62]. Endomyocardial biopsies further suggest this, revealing apoptosis of microvascular endothelial cells [63,64]. Notably, microcirculatory dysfunction in TTS is transient, and its recovery correlates with improved myocardial function [10].

Microvascular dysfunction seems to be a secondary phenomenon rather than a primary cause. Indeed, left ventricle (LV) perfusion defects are reversible and emerge only after cardiac dysfunction develops; moreover, the coronary flow reserve at one-year follow-up did not show ongoing microvascular dysfunction among TTS [65,66].

Positron-emission tomography studies showed hyperperfusion in basal LV segments rather than ischemia in affected regions [48], and experimental models showed contractile dysfunction precedes perfusion changes [52].

In some TTS patients, left ventricular outflow tract obstruction (LVOTO) has been observed. LVOTO may contribute to myocardial dysfunction that could potentially cause cardiogenic shock [35,67]. Midcavity obstruction, potentially due to excessive sympathetic stimulation, could lead to apical subendocardial ischemia, causing ballooning [35]. However, the presence of apical-sparing patterns in many TTS cases suggests that it is not the primary cause [68,69]. Additionally, the right ventricular involvement in many TTS patients cannot be explained by LVOTO alone, suggesting that LVOTO is more of a complication than a central cause [70].

## 4. The Role of Oxidative Stress in the Pathophysiology of Takotsubo Syndrome

Oxidative stress has a critical role in the pathogenesis of TTS due to its detrimental effects on both endothelial and myocardial function. This connection has been highlighted by multiple cellular [71,72], pre-clinical [40,73,74,75,76,77], and clinical studies [41,78,79,80]. Refs. [40,41,73,74,75,76,78,79,80] found that during the acute phase of TTS, the sudden release of catecholamines amplifies ROS production through adrenergic receptor stimulation [81], leading to myocardial injury and the characteristic left ventricular dysfunction observed in TTS patients [72,78].

In response to elevated ROS levels, several protective antioxidant mechanisms, including the upregulation of the Nuclear Factor Erythroid 2-related Factor 2 (Nrf2) pathway, are activated. This pathway enhances the expression of antioxidant genes such as superoxide dismutase (SOD), catalase, and glutathione peroxidase (GPX), which help neutralize ROS and mitigate myocardial damage [72,82]. When insufficient protective mechanisms are used to counteract the overwhelming oxidative stress, myocardial injury is sustained [76,82].

Moreover, myocardial biopsies from acute TTS patients show increased superoxide production, providing direct evidence of oxidative stress-related myocardial injury [72].

Mitochondrial dysfunction has a central role in oxidative stress, contributing to impaired myocardial energy metabolism and contractile dysfunction, particularly in ischemia and excessive catecholamine stimulation, such as TTS. Indeed, stress-induced catecholamine surges can lead to mitochondrial damage, resulting in reduced ATP production and an imbalance in ROS homeostasis. This dysfunction compromises cardiomyocyte viability and function, exacerbating the transient myocardial stunning characteristic of TTS. Additionally, mitochondrial impairment may activate apoptotic pathways and inflammatory responses, further worsening myocardial injury [77,83,84].

Oxidative stress may impact TTS pathophysiology by endothelial dysfunction, which is caused by the disruption of endothelial nitric oxide synthase (eNOS), an enzyme essential for maintaining vascular function [46]. In healthy individuals, eNOS produces nitric oxide (NO), which supports vasodilation, preserves endothelial integrity, and promotes vascular homeostasis [85]. NO also helps regulate blood vessel tone, prevent thrombosis, and exert anti-inflammatory effects, all contributing to cardiovascular health [86]. However, in TTS, this process is impaired due to the depletion of tetrahydrobiopterin (BH4), a crucial cofactor for eNOS activity [87]. Without sufficient BH4, eNOS becomes uncoupled and generates ROS instead of NO, disrupting the balance between vasodilation and vasoconstriction [88,89]. The ROS accumulation leads to endothelial dysfunction, exacerbates myocardial injury, and contributes to microcirculatory impairment and myocardial stunning, hallmarks of TTS [85,88].

Inflammation is another key factor in TTS pathophysiology. Elevated levels of C-reactive protein (CRP) and increased leukocyte counts, markers of systemic inflammation, are commonly observed in TTS patients [87,90,91]. Myocardial biopsies reveal substantial macrophage infiltration within the myocardium, with activated macrophages generating ROS via the nicotinamide adenine dinucleotide phosphate (NADPH) oxidase complex. This further contributes to both endothelial and myocardial dysfunction [83].

**Figure 2:**
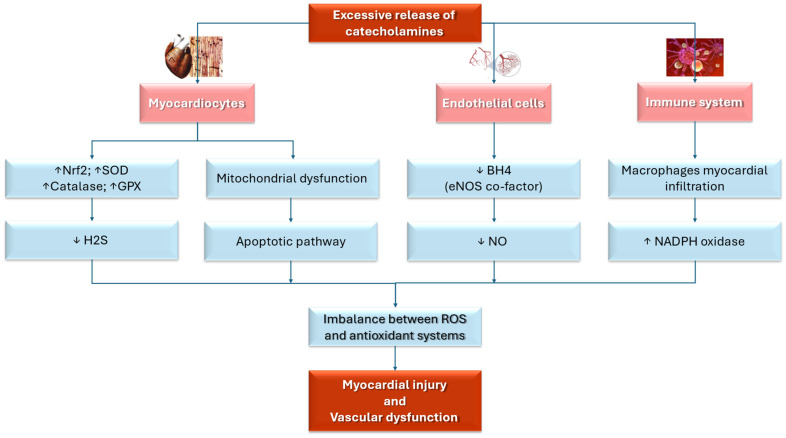
This illustration summarizes the mechanisms that link oxidative stress and TTS. BH4: tetrahydrobiopterin; eNOS: endothelial nitric oxide synthase; GPX: glutathione peroxidase; H2S: hydrogen sulfide; NADPH: nicotinamide adenine dinucleotide phosphate; NO: nitric oxide; NrF2: Nuclear Factor Erythroid 2-related Factor 2; SOD: superoxide dismutase; ROS: reactive oxygen species; ↑ increase; ↓: decrease. Red: principal triggers and targets. Pink: cells target. Blue: physiopathological and molecular mechanisms.

Finally, oxidative stress is not only a central factor in the acute phase of TTS but also has a key role in the recovery process, highlighting its fundamental role in the disease’s pathophysiology. Indeed, oxidative stress and inflammation might remain active during the recovery phase of TTS, prolonging myocardial dysfunction. Myocardial edema, detected by cardiac magnetic resonance imaging (CMR), may persist for months after the acute event, even in the absence of significant coronary perfusion deficits [45]. The ongoing presence of macrophages in the myocardium of patients with recent-onset TTS further amplifies ROS production, delaying recovery and prolonging oxidative damage [74]. Figure 2 summarizes the role of oxidative stress in TTS.

## 5. Studies Supporting the Role of Oxidative Stress in Takotsubo Syndrome

Several pre-clinical [42,74,75,76] and clinical [41,78,79,80] studies support the role of oxidative stress in TTS. Table 1 and Table 2 summarize the features of pre-clinical and clinical studies.

In a female rat model, Surikow et al. [42] showed TTS-like echocardiographic changes, including apical ballooning, after isoprenaline (ISO) administration. Nitrosative stress and Poly (ADP-ribose) polymerase-1 (PARP-1) activation seemed to be involved in the TTS genesis since the pretreatment with 3-amino benzamide, a PARP-1 inhibitor, effectively reduced the OS-negative inotropic effects [42]. However, there are several limitations to the current study, beginning with those inherent in an animal model. Therefore, the use of PARP-1 inhibitors in men should be supported by clinical data, considering dosage significance and translational discrepancies.

In a murine model of TTS using immobilization-stressed rats, Ueyama et al. [75] found an upregulation of heme oxygenase-1 (HO-1) in cardiac and aortic macrophages, a well-recognized marker of oxidative stress. The blockade of alpha- and beta-adrenoceptors significantly attenuated the stress-induced increase in HO-1 mRNA levels in the heart [75].

Additionally, in ISO-induced TTS-rats, Mao et al. [74] revealed an upregulation of the phosphatidylinositol 3-kinase (PI3K)/protein kinase B (AKT)/mammalian target of rapamycin (mTOR) pathway, which, in turn, caused oxidative stress (as assessed by enhanced levels of dihydroethidium) and apoptosis. The inhibition of AKT significantly reduced the cardiac injury induced by ISO treatment, thus suggesting the role of oxidative stress and the PI3K/AKT/mTOR activation in TTS genesis [74].

In a murine model of TTS induced by ISO, Zhang et al. [76] observed reduced myocardial and circulating hydrogen sulfide (H2S) levels, a well-known endogenous antioxidant agent [92,93], and an increased expression of NADPH oxidase subunits. Treatment with sodium hydrosulfide (NaHS) effectively attenuated cardiac injury, alleviated OS, and normalized the expression of NADPH oxidase subunits.

In a prospective observational study by Nguyen et al. [41] including 56 TTS patients (vs. 81 aged-matched controls), the TTS group showed significantly lower levels of asymmetric dimethylarginine (ADMA) and a greater increase in platelet responsiveness to NO (p = 0.0001), both acutely and after 3 months. These results suggest that TTS has higher NO signaling compared to controls.

In a case series of 3 TTS female patients, Nef et al. [78] performed a systematic DNA expression profiling of cardiac genes through microarray analysis, assessing both the acute and recovery phases. TTS patients showed an upregulation of Nrf2 genes in response to oxidative stress. Nrf2 is a pivotal regulator of a wide array of antioxidant enzymes, acting as a powerful endogenous signaling pathway that protects against oxidative stress-induced endothelial dysfunction [94].

In a prospective observational study by Nanno et al., including 8 patients with TTS and 8 age-matched patients with AMI, TTS patients showed higher circulating norepinephrine and urinary 8-hydroxy-2′-deoxyguanosine (8-OHdG) levels. Among TTS patients, the improvement in ventricular dysfunction in TTS was improved in parallel with a decrease in U-8OHdG within 2 weeks. Indeed, cardiac sympathetic hyperactivity and myocardial oxidative stress were more pronounced in TTS cardiomyopathy than in ischemic heart disease [79].

In a prospective observational study by Scally et al. [80], including 55 patients with TTS and 51 age-, sex-, and co-morbidity-matched controls, TTS patients, evaluated by cardiac magnetic resonance imaging with ultrasmall superparamagnetic particles of iron oxide, showed USPIO enhancement and higher serum level of systemic proinflammatory cytokines during the acute phase, which remained elevated at five months follow-up.

In summary, all this evidence suggests the key role of OS and inflammation in the pathogenesis of TTS cardiomyopathy [87].

## 6. Treatment Strategies in Takotsubo Syndrome

The pharmacological treatment of TTS is still debated due to the lack of evidence from randomized clinical trials. To date, the data present in the literature are characterized by several limitations for the study design and sample size. Therefore, no specific medical treatment has been demonstrated to protect against in-hospital mortality and to promote long-term survival in TTS [11].

There are contrasting data about the use of beta-blockers (BBs) both in the acute and chronic phases of the disease. Table 3 summarizes the clinical trials regarding the role of BBs in TTS.

The preadmission beta-blocker therapy did not seem to protect against all causes of mortality, and indeed, it appeared to be associated with a 4.5-fold increased risk of adverse cardiac events [96] in a prospective observational study by Topf et al. [95], including 56 TTS patients [96]. Similarly, in a retrospective nationwide cohort study by Isogai et al. [96], including 2672 patients, no significant difference in 30-day in-hospital mortality between the early beta-blocker group and controls was observed.

In contrast, in a case series by Santoro et al. [97], including 10 TTS patients with LVOTO, early esmolol infusion effectively reduced intraventricular gradient without adverse effects. TTS patients discharged on BBs had a lower risk of all-cause death (adjusted HR: 0.56; 95% CI: 0.36–0.89) and non-cardiac death (adjusted HR:0.56; 95% CI: 0.31–0.89) vs. those not treated with BBs in a prospective multicenter study including 825 TTS patients.

Similar results were found in a cohort of 2853 TTS patients enrolled in the international multicenter GEIST registry (The German Italian Spanish Takotsubo Registry); TTS patients discharged on BBs showed a lower mortality rate (hazard ratio [HR]: 0.71; 95% CI: 0.55–0.90) compared to those discharged without BBs at admission, particularly within the first year post-discharge [98].

BB therapy was not associated with a lower risk of TTS recurrence consistent with previous findings [99].

In contrast, in a registry of 1750 TTS patients by Templin et al. [3], BB treatment was not associated with a long-term survival benefit. However, in the same registry, the use of angiotensin-converting enzyme inhibitors (ACE-i) or angiotensin-receptor blockers (ARBs) seems to effectively improve survival at 1 year [3] and reduce the risk of recurrence.

Further, a meta-analysis by Santoro et al. showed that βBs, ACEIs/ARBs, statins, and aspirin did not significantly reduce recurrences of TTS [100].

In addition, early use of intravenous N-acetylcysteine (NAC), followed by/or oral ramipril for 12 weeks [101], was evaluated in an ongoing multicenter, randomized, placebo-controlled trial, the N-AcetylCysteine and RAMipril in Takotsubo Syndrome Trial (NACRAM). The main outcomes evaluated include myocardial edema resolution on cardiac magnetic resonance imaging, enhanced left ventricular systolic function assessed by global longitudinal strain on echocardiography, improved quality of life, and changes in inflammatory markers. These agents have been utilized primarily to reduce nitrosative stress [101].

Given the established role of ROS in TTS pathogenesis, regulating ROS—particularly within mitochondria—could represent a promising therapeutic strategy in TTS [87]. Several drugs are being evaluated regarding their antioxidant effect [71,102,103].

In this context, several studies have emphasized the anti-inflammatory and antioxidant effects of SGLT2 inhibitors in experimental models of cardiac disease [104,105]. In a rat model of ISO-induced TTS by Tatarcheh et al. [71], treatment with empagliflozin effectively reduced the occurrence and mitigated the progression of TTS. Moreover, ROS levels in ISO rats were normalized after empagliflozin therapy. These findings suggest that SGLT2 inhibitors could be a promising therapeutic target for TTS [48].

PARP-1 inhibitors, known for their impact on nitrosative stress, exogenous treatment with sodium hydrosulfide could be an alternative therapeutic option for TTS, but clinical trials investigating their effects remain unsettled [42].

The administration of α-lipoic acid (ALA) may be useful to improve myocardial bioenergetics and reduce levels of CRP, tumor necrosis factor α (TNF-α), and nitro-tyrosine. In a randomized controlled trial by Marfella et al. [102], including 48 TTS patients, the administration of α-lipoic acid (ALA) vs. placebo was evaluated. In detail, patients underwent perfusion myocardial scintigraphy with technetium 99 m at hospital admission and at 12 months after the acute event. Routine analysis, oxidative stress serum markers, and proinflammatory cytokines were also assessed. The ALA group showed a greater reduction in iodine 123 meta-iodobenzyl guanidine left ventricular uptake defect compared to placebo. Additionally, nitro-tyrosine expression differed significantly at baseline and at the 12-month follow-up in the ALA group but not in the placebo. However, the clinical benefits of ALA treatment remain uncertain [102].

Statins have been studied in TTS patients in the GEIST registry [103]. The author evaluated 2429 consecutive TTS patients, of whom 1293 (53.2%) were discharged on statins, while 1136 (46.8%) were not. However, survival analysis revealed no significant difference in mortality rates between the groups. Therefore, statin therapy following a TTS event was not associated with improved long-term prognosis [103].

In summary, the current scientific landscape regarding the use of molecules with antioxidant effects in the treatment of TTS is characterized by the limited availability of data in the literature, which are often conflicting, and by the complete absence of randomized and controlled clinical trials that support their use in clinical practice. Future research should prioritize the identification and validation of specific, reliable biomarkers of oxidative stress in TTS. This includes investigating markers of specific ROS species (e.g., superoxide, hydrogen peroxide) and antioxidant enzyme activity (e.g., SOD, catalase, GPX). The establishment of such biomarkers would significantly enhance diagnostic accuracy, enable more precise risk stratification, and facilitate the monitoring of treatment response. Furthermore, there is a compelling need for studies focused on phenotyping TTS patients based on their distinct oxidative stress profiles. This approach could elucidate the observed variability in clinical presentation, prognosis, and therapeutic responsiveness. Finally, longitudinal studies are essential to fully characterize the long-term trajectory of oxidative stress in TTS and to rigorously evaluate the potential of antioxidant therapies in preventing adverse cardiac remodeling and improving long-term clinical outcomes.

## 7. Conclusions

Oxidative stress seems to play a pivotal role in the pathogenesis of TTS, functioning as an intermediary between several causal mechanisms and the clinical manifestation of the disease. In TTS, despite activating protective antioxidant pathways, the excessive oxidative stress during the acute phase overwhelms the body’s defense mechanisms, contributing to persistent myocardial injury. These findings highlight the critical role of oxidative stress in TTS and suggest that targeting oxidative damage may provide a promising therapeutic approach for improving outcomes in TTS patients. To date, studies in the literature correlate the possibility of using oxidative stress as a therapeutic target, but there are still few data available. Therefore, specific future research avenues are needed that can validate the use of oxidative stress biomarkers in TTS patients and the application of therapeutic approaches. Antioxidant therapies might be particularly beneficial in TTS patients with evidence of persistent inflammation, impaired microvascular function, or those at high risk for recurrent events.

## Figures and Tables

**Table 1:** The principal features of pre-clinical studies regarding the linkage between oxidative stress and Takotsubo Syndrome.

Author	Years	Model of Cohort	Primary Oxidative-Stress Marker	Major Result
Surikow et al. [40]	2015	Pre-clinical	3-Nitrotyrosine	↑ 3-Nitrotyrosine
			Poly(ADP-ribose)	↑ Poly(ADP-ribose)
Willis et al. [77]	2015	Pre-clinical	Reactive oxygen species	↑ Diastolic RyR2 activity
Wu et al. [73]	2023	Pre-clinical	Malondiadehide	↑ Malondiadehide
			NOX2	↑ NOX2
			NOX4	↑ NOX4
Mao et al. [74]	2020	Pre-clinical	Reactive oxygen species	↑ Reactive oxygen species
			Mitochondrial superoxide generation	↑ Mitochondrial superoxide generation
Ueyama et al. [75]	2009	Pre-clinical	Oxygenase-1	↑ Oxygenase-1
Zhang et al. [76]	2017	Pre-clinical	NOX4	↑ NOX4
			p67	↑ p67
			Hydrogen sulfide	↓ Hydrogen sulfide

ADP: adenosine diphosphate; NOX: nitrous oxide. ↑: increase; ↓: decrease.

**Table 2:** The principal features of clinical studies regarding the linkage between oxidative stress and Takotsubo Syndrome.

Author	Years	Model of Cohort	Primary Oxidative-Stress Marker	Major Result
Nguyen et al. [41]	2013	56 patients	Nitric oxide	↑ Nitric oxide
			Asymmetric dimethylarginine	↓ Asymmetric dimethylarginine
Nef et al. [78]	2008	3 patients	Nuclear factor erythroid 2-related factor 2	↑ Nuclear factor erythroid 2-related factor 2
Nanno et al. [79]	2015	16 patients	Plasma catecholamines	↑Plasma catecholamines
			Urinary 8-hydroxy-2′-deoxyguanosine	↑Urinary 8-hydroxy-2′-deoxyguanosine
Scally et al. [80]	2019	106 patients	Ultrasmall superparamagnetic particles of iron oxide	↑Ultrasmall superparamagnetic particles of iron oxide

↑: increase; ↓: decrease.

**Table 3:** Clinical trials about the role of BBs in TTS.

Author and Years	Research Methods	Sample Sizes (N)	Principal Outcomes	Results
Templin et al. [3] 2015	Retrospective study	1750	Rate of mortality during one year of follow-up	No evidence of any survival benefit from the use of BBs (P = 0.53)
Topf et al. [95] 2022	Prospective study	56	Occurrence of hemodynamically relevant arrhythmia, cardiac decompensation, and all-cause adverse cardiac events during hospitalization during 4 years of follow-up	Increased risk of all-cause complications relative to patients without BBs in preadmission medication (52.0% vs. 19.4%, p = 0.010; OR 4.5 (95% Cl 1.38–14.80))
Isogai et al. [96] 2016	Retrospective study	2672	Thirty-day in-hospital mortality was compared between patients who started BBs therapy on hospitalization day 1 or 2 (early β blocker group) and those who did not receive BBs during hospitalization (control group)	There was no significant difference in 30-day in-hospital mortality between the early BBs group and control group (2.4% vs. 2.0%, p = 0.703; risk difference, 0.4%; 95% CI, −1.2% to 2.0%)
Santoro et al. [97] 2016	Prospective study	96	Evaluation of the hemodynamic effects, safety, and feasibility of selective BBs (β1) with a short half-life, esmolol	LVOT pressure gradient before treatment was 47.6 ± 16.6 mmHg and after 18.2 ± 2.3 mmHg (P = 0.0091)
Raposeiras-Roubin et al. [98] 2025	Prospective study	2853	Assessing the impact of beta-blocker therapy on long-term mortality and TTS recurrence	TTS patients discharged on BBs showed a lower mortality rate (HR: 0.71; 95% CI: 0.55–0.90) compared to those discharged without BBs at admission, during a mean follow-up of 2.6 years, particularly within the first-year post-discharge

BBs: bet-blockers; CI: confidence interval; HR: hazard ratio; LVOT: left ventricle outflow tract; OR: odds ratio; TTS: Takotsubo syndrome.

## Data Availability

No new data were created or analyzed in this study. Data sharing is not applicable to this article.

## Abbreviations

The following abbreviations are used in this manuscript:

8-OHdG	8-hydroxy-2′-deoxyguanosine
ACE-i	Angiotensin-converting-enzyme inhibitors
ACS	Acute coronary Syndrome
ADMA	Asymmetric dimethylarginine
ALA	α-lipoic acid
AKT	Protein kinase B
AMI	Acute myocardial infarction
ARBs	Angiotensin-receptor blockers
BH4	Tetrahydrobiopterin
CFR	Coronary flow reserve
CRP	C-reactive protein
ECG	Electrocardiogram
eNOS	endothelial nitric oxide synthase
GEIST	GErman Italian Spanish Takotsubo
GPX	Glutathione peroxidase
H2S	Hydrogen Sulfide
HO-1	Heme oxygenase-1
HPA	Hypothalamic-pituitary-adrenal
ISO	Isoprenalin
LVEF	Left ventricular ejection fraction
LVOTO	Left ventricular outflow tract obstruction
LVWMAs	Left ventricular wall motion abnormalities
MCE	Myocardial contrast echocardiography
Mito-ROS	Mitochondrial-derived ROS
mTOR	Mammalian target of rapamycin
SGLT2	Sodium/glucose cotransporter 2
NACRAM	N-AcetylCysteine and RAMipril in Takotsubo Syndrome Trial
NADPH	Nicotinamide adenine dinucleotide phosphate
NaHS	Sodium hydrosulfide
NO	Nitric oxide
NOX	NADPH oxidase
Nrf-2	Nuclear factor erythroid 2-related factor 2
PARP-1	Nitrosative stress and Poly (ADP-ribose) polymerase-1
PI3K	Phosphatidylinositol 3-kinase
ROS	Reactive oxygen species
SH	Sodium hydrosulfide
SNS	Sympathetic nervous system
SOD	Superoxide dismutase
TNF-α	Tumor necrosis factor α
TTS	Takotsubo Syndrome
TIMI	Thrombolysis in Myocardial Infarction
USPIO	Superparamagnetic particles of iron oxide
WMAs	Wall motion abnormalities
